# Descending Necrotizing Mediastinitis of Oropharyngeal Infections

**DOI:** 10.5681/joddd.2009.021

**Published:** 2009-09-16

**Authors:** Mohsen Sokouti, Saeed Nezafati

**Affiliations:** ^1^Associate Professor, Department of Thoracic Surgery, Faculty of Medicine and Imam Reza Hospital, Tabriz University of Medical Sciences, Tabriz, Iran; ^2^Assistant Professor, Department of Oral and Maxillofacial Surgery, Faculty of Dentistry, Tabriz University of Medical Sciences, Tabriz, Iran

**Keywords:** Abscess, oral surgery, oropharyngeal

## Abstract

**Background and aims:**

Descending necrotizing mediastinitis (DNM) is a rare and life-threatening infection. Management of this condition is very difficult and before 1990s, DNM had a mortality rate of 40% despite the use of antibiotics. One of the etiologies of this condition is rapid downward spread of oropharyngeal infection along the cervical fascia planes into the medi-astinum.

**Materials and methods:**

Patients with DNM from odontogenic, peritonsillar and retropharyngeal origins, who underwent surgical treatment from 1990 to 2007, were reviewed. Data extracted from medical records of the patients included age, gender, origin of the infection, surgical approaches, and the cause of mortality. Descriptive data were expressed as a Mean ± SE.

**Results:**

Thirteen patients aged 15 to 56 (mean, 34.5 years old; 8 males and 5 females) were studied. The origins of infection included odontogenic abscess in 10 cases and peritonsillar and retropharyngeal abscess in 3 patients. The mean duration from onset of symptoms to the surgery was 12.18 ± 0.98 days (range 3 to 24 days) and the mean duration from initial surgery to dis-charges was 28.51 ± 3.25 days (range 5 to 92 days). Post-operative mortality was seen in three patients.

**Conclusion:**

Descending necrotizing mediastinitis can arise from odontogenic abscesses and must be detected as early as possible, as it is a life-threatening infection.

## Introduction

One rare, important emergent condition in head and neck infections is descending necrotizing mediastinitis (DNM). It has a high mortality rate, mainly due to rapid spread of the polymicrobial infection and delay in diagnosis and treatment. DNM may begin from acute infection of mediastinal structures as a result of rupture and disease of esophagus and tracheobronchial trees or may be related to the spread of oropharyngeal and odontogenic infections to the mediastinum, pleural spaces, pericardium and even to the abdomen through contagious deep spaces of the neck and mediastinum.^1 , 2^ The diagnostic criteria of DNM include evidence of severe oropharyngeal infection, clinical or radiological signs and symptoms of mediastinal infections, and relationship between DNM and oropharyngeal infection.^3^

Before 1990s, the mortality rate of DNM with aggressive surgical techniques was about 40-50% due to the persistence of infection with empyema, pleural and pericardial effusions, pericarditis, and blood vessel erosion.^3 - 5^ After 1990s with the use of aggressive surgical techniques, the mortality rate decreased to 15.4%. In the presence of severe illness, the mortality rate may be as high as 67%.

Most authors have recommended early diagnosis and effective surgical drainage. Brunelli et al^6^ recommend the aggressive approaches like thoracotomy, when the spread of infection reaches below carina. In this article, we report our experiences in the treatment of patients with DNM using aggressive surgical techniques like radical debridment of the cervical fascia, mediastinum, and both pleural cavities. To the best of our knowledge, this is the third largest series reported after Estrera et al^3^ and Freeman et al.^7^

## Materials and Methods

In this retrospective study, patients who underwent surgical drainage of DNM originated from oropharyngeal infections between 1990 and 2007 were reviewed. Data of 13 patients were extracted from medical records of the hospital. Of these, 10 patients had DNM secondary to oropharyngeal infections and three had peritonsillar and retropharyngeal abscesses. Patients with DNM arising from esophageal perforation were not included in this study because of the different pathogenesis and treatment.

Data obtained from medical records included age, gender, origin of the infection, surgical techniques, progress of the disease, outcome of the treatment, and the cause of death. All patients were diagnosed according to clinical and radiographic signs. Preoperative cervical and thoracic computed tomography (CT) scans were performed in eight patients diagnosed with cervical abscess with DNM. Broad-spectrum antibiotics were initiated for all patients and changed according to the antibiogram if it was necessary.

Surgery was performed under general anesthesia. Patients were positioned supine with neck extended and tilted to the non-operating side. All of the cervical abscesses were drained with oblique incision parallel to the border of the sternocleidomastoid muscle, and exploration of the neck and superior mediastinum were performed if it was necessary. The exploration continued to peritracheal, periesophageal and perivascular spaces up to down. The submandibular abscesses drained separately and tunneled to the cervical spaces. Necrotic materials were debrided and drained with one to three Penrose drains. In cases of empyema, pus was drained with one or two closed tube drainages. Eight patients had undergone pleuromediastinal drainage and debridment of mediastinal tissues and decortication of pleura. Eleven patients, in whom the extent of mediastinitis had reached to superior mediastinum, were treated with cervical and mediastinal drainage via cervical incisions. In seven patients with submandibular and suprahyoid abscess, submandibular drainage was performed by an oral and maxillofacial surgeon.

The treatment outcomes were evaluated by routine chest x-ray. When extensive mediastinitis and multi-abscess of pleural cavities were proved and diagnosed by computed tomography, thoracotomy and decortication of the thickened pleura and drainage of the mediastinum were performed via a long incision on mediastinal pleura. After drainage and irrigation of the retained pleural cavities, two chest tubes were inserted to the pleural spaces. All patients were transferred to the intensive care unit after the surgery.

### Statistical analysis

The primary endpoint was assessment of death in DNM. Descriptive data were expressed as a Mean ± SE. The statistical package for social science (SPSS 13) was used for statistical analysis.

## Results

Thirteen patients, 8 males and 5 females, aged 15 to 56 years old (mean age, 34.5) were studied retrospectively. A demographic feature of the patients is illustrated in Table 1. The infections were of odontogenic origin in 10 patients and retropharyngeal and peritonsillar abscesses in 3 patients. Symptoms included trismus (n = 11), odynophagia (n = 11), pyrexia (n = 12), pharyngitis (n = 6), cervical bulging and cellulites (n = 12), submandibular abscess sign (n = 7), and respiratory signs (n = 8). Mean time from onset of symptoms until surgery was 12.18 ± 0.98 days (range 3 to 24 days). All patients had polymicrobial, aerobic and anaerobic infections. In 9 patients contra-lateral abscess was detected by cervical CT scan. Eight patients required thoracotomy in one or two sides, decortication, drainage of multipocket abscesses of pleural spaces and pericardiostomy. In the remaining patients with submandibular abscess, one- or two-side cervical drainage, and cervical mediastinotomy was performed. In four patients postoperative CT scan showed residual abscess infections in pleural or mediastinal spaces. These patients underwent right thoracotomy or added with left thoracotomy to explore and drainage of pus cavities of pleura and mediastinum with decortication (Table 2). The mean duration from initial surgery to discharge was 28.51 ± 3.25 days (range 5 to 92 days), and chest tubes were continued for 25 ± 3.85 days. Antibiotic was administered for 40 ± 4.15 days and WBC normalizations were found 9.7 ± 3.21 days after the initial operation. There were three postoperative deaths. Patient number 10 was in septic shock preoperatively and died due to massive aspiration of pus and septic shock on day 1 post-operatively. Two other patients (number 7 & 12) died because of acute respiratory distress syndrome (ARDS), septic shock and multiorgan failure after empyema and mediastinitis.

**Table 1:** Summary of the studied patients’ medical records with descending necrotizing mediastinitis

Patient No.	Sex	Age	Cause	Side	Extent of infection	Kind of surgery	Outcome	Days in ICU
1	F	48	DNM-odontogenic	Right	BCS+ ASPS	BCD-CMD	Discharge	3
2	F	53	DNM-odontogenic	Right	BCS +ASPS+ LE	BCD + LTD + CMD + SMD	Discharge	4
3	M	23	DNM-odontogenic	Left	BCS+ ASPS+LE	BCD + LTD + LTTD + CMD	Discharge	7
4	M	56	DNM-odontogenic	Right	BCS+ ASPS+RE	RCD + RTTD + RTD + CMD + SMD	Discharge	8
5	F	30	DNM-odontogenic	Left	LCS+ RE+ASPS	RCD + RTTR + RTD + CMD + LCD	Discharge	9
6	M	42	DNM-odontogenic	Right	RCS+RE (multiloculated) + ASPS	RCD + RTD	Discharge	4
7	M	36	DNM-odontogenic	Right	BCS+ RE+LE+ ASPS	BCD + RTD + LTD + CMD	Death / ARDS & septic shock	29
8	M	39	DNM-odontogenic	Left	LCS+ ASPS	LCD + CMD + SMD	Discharge	5
9	M	45	DNM-odontogenic	Left	LCS+ ASPS	LCD + CMD + SMD	Discharge	3
10	F	18	DNM-odontogenic	Right	BCS	BCD + SMD	Death / aspiration	—
11	M	15	DNM-peritonsillar+ Retropharyngeal	Right	BCS + ASPS	BCD + CMD + SMD	Discharge	2
12	F	20	DNM-peritonsillar+ Retropharyngeal	Left	BCS + ASPS+ LE + PP	LCD + CMD + LTTD + LTD + Pericardiostomy	Death / septic shock	33
13	M	48	DNM-peritonsillar	Left	LCS + ASPS+ LE+ PP	LCD + CMD + LTTD + LTD + Pericardiostomy	Discharge	17

BCD: Bilateral Cervical drainage; LTD: Left Thoracotomy Decortication; RTTD: Right Thoracostomy Tube Drainage; LTTD: Left Thoracostomy Tube Drainage; RCD: Right Cervical Drainage; LCD: Left Cervical Drainage; CMD: Cervical Mediastenal Drainage via cervical incision; SMD: Sub Mandibular Drainage; BCS: Bilateral Cervical Space; ASPS: Anterosuperior and Posterior Mediastenal Space; RE: Right Empyema; LE: Left Empyema;BE: Bilateral Empyema; LCS: Left Cervical Space; PP: Purulent Pericarditis; ARDS: Acute Respiratory Distress Syndrome.

**Table 2:** Reasons of pleuromediastinal drainage via thoracostomy or cervicotomy in descending necrotizing mediastinitis

Reasons	Frequency
Combined lung abscess and empyema	1
Right multiloculated empyema	5
Bilateral empyema	4
Left multiloculated empyema	4
Purulent mediastinitis and unruptured mediastinal pleura	4
Closed tube thoracostomy drainage	2
Cervical mediastinotomy	4
Thoracotomy with percardiostomy	2

## Discussion

Descending necrotizing mediastinitis (DNM) is an acute and polymicrobial infection extending downward from the oropharynx. Our report consists of mediastinitis cases due to the cervical and mediastinal spread of oropharyngeal infection with pus descending to the mediastinum. The incidence of DNM is low, but the mortality rate is very high. Reports of large series of patients with DNM are rare in the literature. This is the third largest series, to the best of our knowledge, reported after Estrera et al^3^ and Freeman et al.^7^

The formation of an oropharyngeal abscess as a consequence of dental or tonsillar infection can lead to the development of DNM.^3^ Oropharyngeal infections usually spread to the mediastinum through loose anatomical structures of the pretracheal, perivascular, and retropharyngeal spaces.^8 , 9^ According to the effect of gravity and the negative pressure of mediastinum and pleural cavities, rapid spread of infection is facilitated by tissue necrosis. In our study, odontogenic and peritonsillar infections led to DNM in 10 and 3 patients, respectively. In its severe clinical form, the mortality rate of DNM remains high, despite the availability of broad-spectrum antibiotics and the development of surgical and intensive care techniques.^10^

Management of DNM includes early diagnosis, adequate antibiotics, and effective surgical drainage of the pus and debridment of infected tissues. According to the classification of Endo et al,^11^ DNM is divided into three types (I, IIA, IIB). Different surgical treatments have been suggested according to the extent of infection based on this classification: Patients of type I DNM might not always require aggressive mediastinal drainage. Diffuse DNM (type IIB) demands complete mediastinal drainage with debridment through thoracotomy. In type IIA, subxiphoid mediastinal drainage without sternotomy is adequate. Many authors have reported the advantage of drainage and debridment through more aggressive open surgical approaches in the management of patients with DNM.^12 , 13^ However, these aggressive approaches may worsen the condition of the patients with sepsis and also increase the length of hospital stay in addition to higher morbidity and mortality. Median sternotomy for exposure of both hemithoraces and all spaces of mediastinum may cause subsequent osteomylitis and dehiscence of the sternum. Ris et al^13^ suggest clamshell incisions but do not use it in unstable and critically ill patients. In the present study, 8 patients of type IIB, underwent cervical drainage with pleuromediastinal draining by thoracostomy and thoracotomy. Postoperative complications such as nosocomial aspirated pneumonia occurred in three, and empyema in 8 patients treated with thoracotomy and decortication. These patients recovered. However, one of the patients died after cervical drainage because of aspiration of large amount of pus.

The organisms in DNM are typically highly-virulent mixed aerobes and anaerobes often acting synergistically.^14 , 15^ Because patients with DNM have usually been treated with broad-spectrum antibiotics emergently before performing antibiogram, cultures are often negative. On the basis of antibiogram in our study, two patients suffered from uncontrolled sepsis of mixed anaerobic infections.

There were three cases (23%) of postoperative death in our series, whereas 25-40% patients with DNM in the literature are reported to have died during the course of treatment.^10^

Two patients underwent surgical drainage through thoracotomy for residual multiabscess pockets and decortications, but sepsis could not be controlled and they expired on days 29 and 33 after initial operation. The causes of death were acute respiratory distress syndrome or multiorgan failure. We think these mortalities were not related to ineffectiveness of pleuromediastinal drainage but related to the severity of infection and aspiration. Serial transcervical and transthoracic procedures have been recommended if necessary. The need for tracheostomy should be assessed on an individual basis for patients with DNM,^7^ as would be needed in patient number 10.

## Conclusion

Descending necrotizing mediastinitis remains a life-threatening infection, which necessitates radical cervicotomy followed by mediastinal drainages. The surgical treatment includes debridment of the cervical, retrovisceral, tracheal fascias and mediatinum, and pleural cavities with one or bilateral decortication through thoracotomies. Pericardial effusions or pus drained to pleural cavities can be treated with pericardiostomy and in some severe cases with pericardiectomy.^15^
